# Monoclonal immunoglobulins on red blood cells: a potential supplementary diagnostic indicator for monoclonal gammopathies

**DOI:** 10.3389/fimmu.2025.1636162

**Published:** 2025-09-12

**Authors:** Benxia Bing, Xiaowei Yang, Chenghua Wang, Liang Xu, Shiqing Cheng, Yun Liu, Qiuhan Wang, Chunjuan Zhai, Bing Zhao, Rong Wang, Jing Sun

**Affiliations:** ^1^ Department of Nephrology, Shandong Provincial Hospital affiliated to Shandong First Medical University, Jinan, Shandong, China; ^2^ Department of Emergency Center, Shandong Provincial Hospital affiliated to Shandong First Medical University, Jinan, Shandong, China; ^3^ Department of Clinical Laboratory, Shandong Provincial Hospital affiliated to Shandong First Medical University, Jinan, Shandong, China; ^4^ Department of Cardiology, Shandong Provincial Hospital affiliated to Shandong First Medical University, Jinan, Shandong, China; ^5^ Department of Nephrology, Qilu Hospital of Shandong University, Jinan, Shandong, China

**Keywords:** monoclonal gammopathy, monoclonal immunoglobulins, capillary electrophoresis and immunosubtraction, red blood cells, western blot

## Abstract

**Introduction:**

Red blood cells (RBCs) have the capability to bind and transport a variety of substances. The aim of this study is to investigate the potential of circulating RBCs as carriers of monoclonal immunoglobulins (M proteins).

**Methods:**

Patients who were newly diagnosed with monoclonal gammopathy in the Nephrology Department of Shandong Provincial Hospital affiliated with Shandong First Medical University from April 2023 to December 2024 were included in this study. Western blots were performed on RBC membrane proteins using primary antibodies against human IgG, IgM, IgA, kappa, and lambda.

**Results:**

Forty-nine patients with monoclonal gammopathy were enrolled in this study. Substantial increases in the amounts of erythrocyte-bound immunoglobulins, characterized by monoclonal properties, were observed. Among the 49 patients, 45 (91.8%) had M proteins detected on RBCs, 38 (77.6%) had abnormal serum capillary electrophoresis and immunosubtraction (CE/IS) results, 36 (75.3%) had abnormal serum free light-chain (sFLC) ratios, and 32 (65.3%) had abnormal serum total light-chain (sTLC) ratios. 53.1% (26/49) of patients with monoclonal gammopathy had concordant results between erythrocyte Western blot and CE/IS. 22.4% (11/49) of patients had negative serum CE/IS results but extrinsic monoclonal immunoprotein bands on RBCs, which were consistent with the findings from sFLC, bone marrow flow cytometry, or renal histopathology. 16.3% (8/49) of patients had abnormal but inconsistent results between serum CE/IS and erythrocyte electrophoresis. Four (8.2%) patients had no or polyclonal immunoprotein bands on RBCs.

**Conclusion:**

Monoclonal immunoglobulins were presented on erythrocytes, which may serve as a supplementary diagnostic indicator for the detection of M proteins.

## 1 Introduction

Monoclonal gammopathy is characterized by the presence of a monoclonal immunoglobulin (M protein) in the plasma, urine, or both. M proteins are produced by clonal proliferation of plasma cells or B lymphocytes and may serve as an indicator of underlying malignancies or as a precursor to malignancy, such as monoclonal gammopathy of uncertain significance (MGUS) and monoclonal gammopathy of renal significance (MGRS) (1, 2). The detection of M protein is a fundamental part of the diagnosis and management of patients with monoclonal gammopathies (3).

A spectrum of laboratory tests has been developed to identify M proteins, including serum and urine protein electrophoresis (SPEP and UPEP), serum and urine immunofixation (sIFE and uIFE), immunosubtraction, serum total light chains (sTLCs), serum free light-chain (sFLC), mass spectrometry, and heavy/light chain (HLC) isotype quantitative measurement (4–6). Although SPEP, IFE, and immunosubtraction are the most widely utilized clinical assays, there is a lack of standardization with considerable variability in the reporting practices among laboratories; especially when the concentration of M protein is low, the detection can be challenging in the presence of high polyclonal immunoglobulin backgrounds that comigrate with the M protein (7). The introduction of quantitative serum assays for immunoglobulin sFLC has strongly increased the sensitivity of laboratory testing strategies and provided an objective quantitative test for identifying monoclonal gammopathies (8, 9). However, the sFLC assay cannot pinpoint the M protein isotype and guide the clone-directed therapy.

Red blood cells (RBCs) are the most abundant type of blood cells, and plenty of evidence has shown that RBCs perform many functions other than oxygen and carbon dioxide transport. RBCs bind pathogens via glycophorin A and regulate nitric oxide bioavailability (10, 11). RBCs bind mitochondrial DNA through Toll-like receptor 9 (TLR9) to maintain quiescence and to prevent lung injury (12). RBCs also bind some amyloid substances, such as amyloid β in Alzheimer’s disease and amylin in type 2 diabetes (13–15). Given the multifaceted roles of RBCs in the binding and transporting of a variety of substances, the aim of this study is to investigate the potential of circulating RBCs as carriers of M proteins.

## 2 Methods

### 2.1 Patients

This was a prospective, single-center study to detect whether monoclonal immunoglobulins were bound to RBCs. Forty-nine patients (age ≥ 18 years) who were newly diagnosed with monoclonal gammopathy or MGRS in the Nephrology Department of Shandong Provincial Hospital affiliated with Shandong First Medical University from April 2023 to December 2024 were included in this study. The clinical data, including age, sex, albumin (ALB), serum creatinine (SCR), hemoglobin, estimated glomerular filtration rate (eGFR), 24h urinary protein, as well as the results of renal biopsy and bone marrow biopsy, were collected. We used the Chronic Kidney Disease Epidemiology Collaboration (CKD-EPI) formula to estimate the eGFR.

We enrolled 40 age- and gender-matched healthy individuals as normal controls, along with five patients with lupus nephritis (LN) and five patients with membranous nephropathy (MN) who were positive for anti-phospholipase A2 receptor (PLA2R) antibodies as disease controls.

Written informed consent was obtained from each patient. The research was conducted in compliance with the Declaration of Helsinki. The study was approved by the local ethics committees of Shandong Provincial Hospital, affiliated with Shandong First Medical University (SZRJJ: NO. 2021-145).

### 2.2 Serum M protein detection

In the local clinical practice, patients admitted to the Nephrology Department with kidney diseases routinely received sTLCs assays, sFLCs assays, and capillary electrophoresis and immunosubtraction (CE/IS) for monoclonal gammopathy screening. sTLCs were analyzed by the N Antisera to Human Immunoglobulin/L-chains kit, using a nephelometric analyzer Dade Behring BN II (Siemens, Germany). Concentrations of κFLC and λFLC were assessed using the Siemens N-Latex FLC kappa (κFLC) and lambda (λFLC) assays (Siemens Healthineers, Germany) according to the manufacturer’s instructions. CE/IS was performed using CAPILLARYS 2 (Capillary System) according to the manufacturer’s instruction. The reference ranges for κFLC and λFLC are 6.7–22.4 and 8.3–27 mg/L, respectively, and the κFLC/λFLC is 0.31–1.56. The reference ranges for κTLC and λTLC are 1.7–3.7 g/L and 0.9–2.1 g/L, respectively, and the κTLC/λTLC is 1.35–2.65.

### 2.3 Extraction of erythrocyte membrane proteins

RBCs were isolated from EDTA-anticoagulated blood immediately after blood collection by centrifugation at 956 g for 10 min at 4°C. After centrifugation, the supernatant (plasma and buffy coat) was carefully aspirated to obtain packed RBCs, which were then washed with phosphate-buffered saline (PBS). Then pre-chilled 10 mmol/L hypotonic Tris-HCl buffer (pH 7.4) was addedto the washed RBCs at a volume ratio of 1:40 and then incubated at 4°C for 2h. This step was followed by centrifugation at 4°C and 15,294 g for 15 min to pellet the erythrocyte membranes. RIPA lysis buffer (Solarbio, Beijing, China) was then introduced to dissolve erythrocyte membranes, and the mixture was incubated on ice for 30 minutes. Thereafter, protein samples were extracted from the supernatant following centrifugation. The protein concentration was determined using a BCA protein analysis kit (Epizyme, Shanghai, China).

### 2.4 Western blot analysis

Erythrocyte membrane proteins were denatured in protein sample loading buffer (denaturing, reducing, 5×) and boiled for 10 min. Subsequently the proteins were electrophoresed with 10% SDS-PAGE and then were transferred to a polyvinyl fluoride membrane (PVDF) membrane (Millipore). Dilutions of the following antibodies were made and cocultured with the membrane overnight at 4°C: rabbit monoclonal anti-human lambda antibody (diluted 1:5000, ab124719, Abcam), rabbit monoclonal anti-human kappa antibody (diluted 1:1000, ab124727, Abcam), rabbit monoclonal anti-human IgG gamma chain antibody (diluted 1:5000, ab133470, Abcam), rabbit monoclonal anti-human IgM heavy chain antibody (diluted 1:1000, ab212201, Abcam), rabbit monoclonal anti-human IgA heavy chain antibody (diluted 1:4000, ab133660, Abcam), and rabbit monoclonal anti-human apolipoprotein E (ApoE) antibody (diluted 1:100, ab52607, Abcam). Then incubate the membrane with HRP-conjugated goat anti-rabbit IgG (diluted 1:10000, SA00001-2, Proteintech) for 1h at room temperature. The ECL system and Bio-Rad electrophoretic image analyzer were used to observe the immune reaction zone after the developer was added.

### 2.5 Statistical analysis

Statistical software SPSS version 22.0 (SPSS, Chicago, IL) was employed for statistical analysis. Quantitative data were expressed as mean ± SD, median and 25th–75th percentile, median and range, or number (%).

## 3 Results

### 3.1 Patient characteristics

Based on the results of CE/IS, sTLC or sFLC ratios, and renal biopsy, 49 patients were enrolled in this study, including 48 with abnormal CE/IS results and/or sTLC and/or sFLC ratios, and 1 with MGRS lacking a circulating M protein. The general characteristics of the patients are shown in **Table 1**. These patients were all newly diagnosed patients with monoclonal gammopathy who had not received systematic treatment. Among the 49 patients, 31 were diagnosed with multiple myeloma (MM), of whom 9 underwent renal biopsies. The renal pathological findings included three cases of light chain amyloidosis, two cases of cast nephropathy, three cases of light chain deposition disease (LCDD), and one case of cryoglobulinemia. Two of the 49 patients were diagnosed with Waldenström’s macroglobulinemia (WM) without renal biopsies. Twelve of the 49 patients were diagnosed with MGRS, which included seven cases of light chain amyloidosis, two cases of LCDD, and one case of light/heavy chain deposition disease where proliferative glomerulonephritis with monoclonal IgG deposits (PGNMID) could not be definitively excluded, as well as two confirmed cases of PGNMID. The remaining four patients were considered to have MGUS. Among them, one patient had abnormal CE/IS results (IgG-kappa) and sTLC ratios without any signs of organ involvement. One patient had abnormal CE/IS results (IgG-lambda) and sTLC ratios with minimal change disease (MCD) diagnosed by renal biopsy. One patient had abnormal CE/IS results (IgM-lambda) but normal ratios of sTLC and sFLC with focal segmental glomerular sclerosis (FSGS) diagnosed by renal biopsy. One patient had abnormal CE/IS results (IgG-lambda) along with abnormal sTLC and sFLC ratios. A renal biopsy was not performed due to her advanced age. However, she was positive for anti-PLA2R antibodies, and her proteinuria responded favorably to rituximab therapy, so a diagnosis of MGUS combined with PLA2R-associated MN was considered.

**Table 1:** General characteristics of the study patients.

Variables	Patients (*n* = 49)
Age, median, range (years)	65 (46–83)
Female sex, *n* (%)	21 (42.9)
SCR, median (interquartile range) (µmol/L)	86 (58.1, 167.7)
eGFR, median (interquartile range) (ml/min/1.73m^2^)	74.3 (34.5, > 90)
Hemoglobin, median (interquartile range) (g/L)	108 (91.5, 125.5)
ALB (mean ± SD) (g/L)	33.9 ± 8.01
24h urine protein (mean ± SD) (g/24h)	5.5 ± 5.85
Serum κTLC/λTLC, median (interquartile range)	1.6 (0.88, 3.59)
Serum κFLC/λFLC, median (interquartile range)	0.58 (0.06, 4.32)
Serum IgG (mean ± SD) (g/L)	9.9 ± 7.5
Serum IgA (mean ± SD) (g/L)	2.1 ± 4.5
Serum IgM (mean ± SD) (g/L)	1.7 ± 5
Monoclonal gammopathy	
MM, n (%)	31 (63.3)
WM, n (%)	2 (4.1)
MGRS, n (%)	12 (24.4)
MGUS, n (%)	4 (8.2)

SCR, serum creatinine; SD, standard deviation; eGFR, estimated glomerular filtration rate; ALB, albumin; κTLC, serum total kappa chain; λTLC, serum total lambda chain; κFLC, free kappa chain; λFLC, free lambda chain; Ig, immunoglobulin; MM, multiple myeloma; WM, Waldenström macroglobulinemia; MGRS, monoclonal gammopathy of renal significance; MGUS, monoclonal gammopathy of uncertain significance.

Reference values: SCR (57−111 µmol/L); eGFR (>90 ml/min/1.73m^2^); Hemoglobin (130−175 g/L); ALB (40−55 g/l); 24h urine protein (0−0.15 g/24h); serum κTLC/λTLC (1.35−2.65); serum κFLC/λFLC (0.31−1.56); Serum IgG (8.6−17.4 g/L); Serum IgA (1.0−4.2 g/L); Serum IgM (0.30−2.2 g/L). All reference ranges are based on local laboratory standards of Shandong Provincial Hospital affiliated to Shandong First Medical University.

### 3.2 Immunoproteins on the surface of RBCs

Previous studies have documented small amounts of immunoglobulins (IgG, IgA, or IgM) coated RBCs in healthy subjects as determined by some measurement methods, such as the agglutination-based direct antiglobulin test (DAT), radioimmunoassay (RIA), and enzyme-linked immunosorbent assay (ELISA) (16–18). In this study, we employed Western blot analysis to identify the immunoproteins, including IgG, IgM, IgA, kappa, and lambda, presented on the surface of RBCs in 40 healthy controls, 5 LN patients, 5 MN patients, and 49 patients with monoclonal gammopathy.

**Figure 1:**
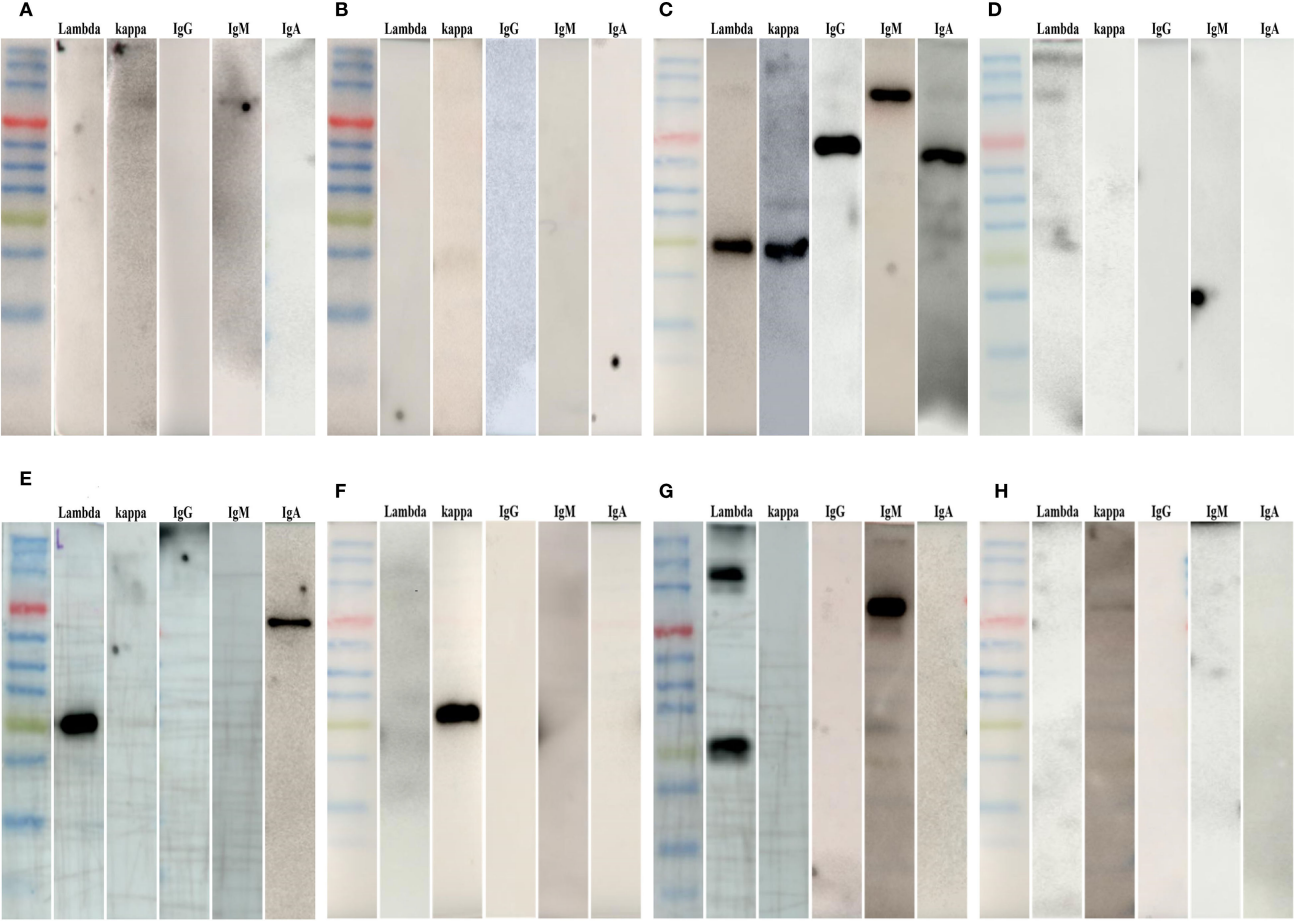
Representative results of erythrocyte electrophoresis. In the Western blot analyses presented, the leftmost column in each image represented the molecular weight marker. Primary antibodies, including rabbit monoclonal antibodies against human lambda, kappa, IgG, IgM, and IgA (arranged from left to right), were utilized in the Western blots. **(A, B)** Representative Western blot results from healthy controls. **(C)** A representative Western blot result from a patient with LN, showing distinct bands for IgG, IgM, IgA, kappa, and lambda. **(D)** A representative Western blot result from a patient with MN who tested positive for anti-PLA2R antibodies, in which no immunoglobulin was detected on erythrocytes. (**E–H**) Representative Western blot results of patients with monoclonal gammopathy.

Among the 40 healthy controls, 4 exhibited very weak bands corresponding to lambda, kappa, IgM, or IgG on RBCs. In five patients with LN, all the tested immunoproteins were present on the RBCs’ surface. That was immunoglobulins binding to RBCs in patients with LN exhibiting polyclonal characteristics, rather than being of monoclonal origin. In five patients with MN who were positive for anti-PLA2R antibodies, no immunoglobulin was detected on RBCs. Among the 49 patients with monoclonal gammopathy enrolled in this study, substantial increases in the amounts of erythrocyte membrane-bound immunoglobulins, characterized by monoclonal properties, were observed in 45 (91.8%) patients. Not only intact monoclonal immunoglobulins but also solely monoclonal immunoglobulin fractions were detected on RBCs. Among the remaining four patients, one patient with light chain amyloidosis had all the tested immunoglobulins detected on RBCs. No immunoglobulin was detected on RBCs in one patient with LCDD, one patient with MGUS (renal pathological type was MCD), and one patient with MM. The representative and detailed results of erythrocyte electrophoresis were respectively illustrated in **Figure 1** and **Supplementary Figures 1** and **2**, respectively.

### 3.3 No ApoE on the surface of RBCs

Previous studies have reported that RBCs carry some amyloid substances, such as amyloid β in Alzheimer’s disease and amylin in type 2 diabetes (13–15). Given that ApoE is a protein usually co-deposited in light chain amyloidosis (19, 20), we performed Western blot analysis to assess the presence of ApoE on RBCs in 10 patients with light chain amyloidosis. Our findings revealed an absence of ApoE on RBCs in all subjects examined (data not shown).

### 3.4 Correlations of serum immunoglobulin and free light chain levels with RBC M protein deposition

**Figure 2:**
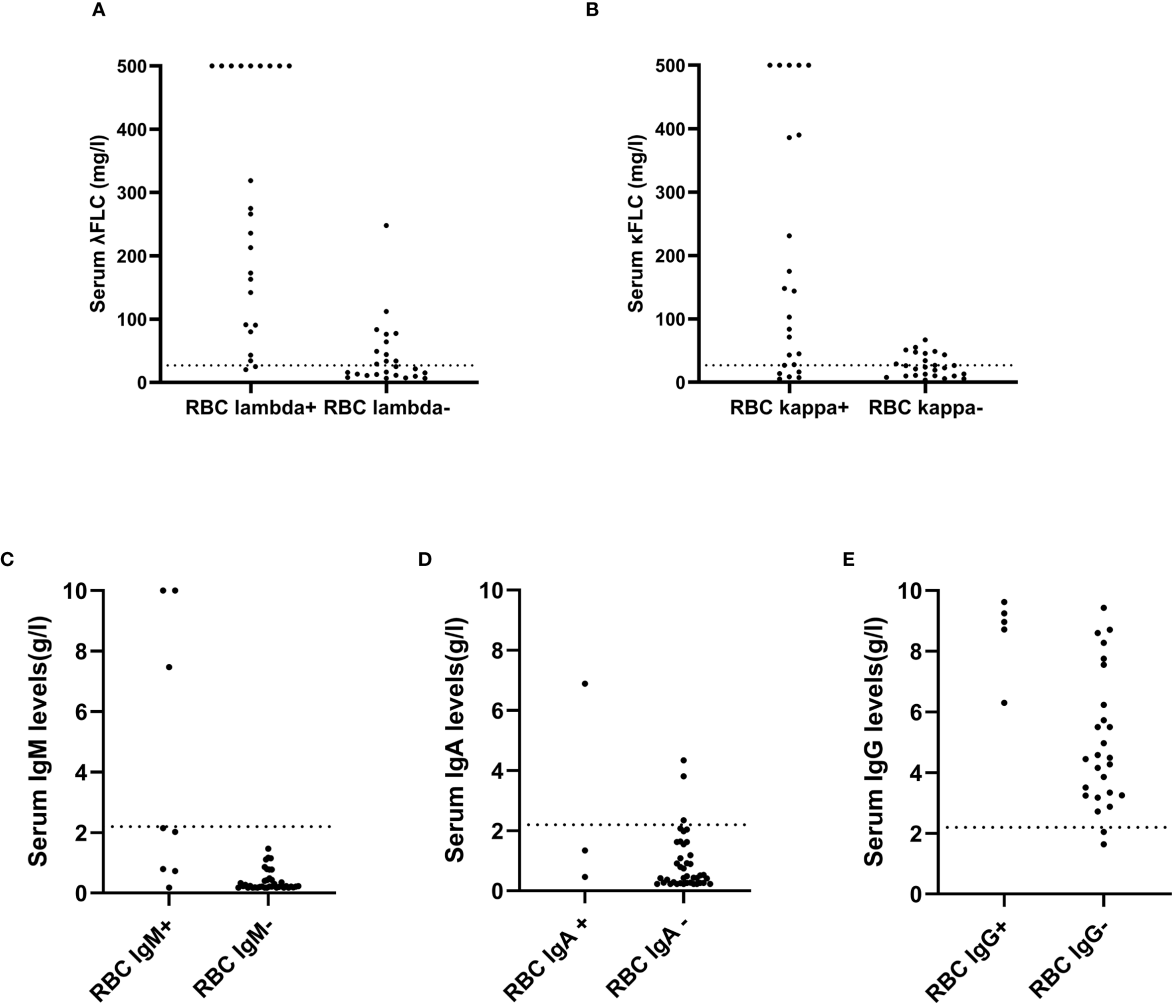
Serum immunoprotein levels in patients with and without the corresponding erythrocyte M proteins. **(A, B)** Serum levels of free light chains. **(C–E)** Serum levels of immnoglobulins. Horizontal dashed lines represented the upper normal limits for the serum levels of free light chains or immunoglobulins.

Subsequently, we explored whether the deposition of M proteins on RBCs was determined by the quantity of serum immunoglobulins. As shown in **Figure 2**, in most patients with M proteins presented on RBCs, the serum levels of the corresponding immunoglobulins were found to be elevated. Nevertheless, it was observed that in a subset of patients with normal serum levels of immunoproteins, the corresponding immunoglobulins were detected on RBCs, and conversely, patients with undetectable RBC M proteins exhibited elevated levels of the corresponding immunoproteins in serum.

### 3.5 Comparisons of erythrocyte electrophoresis and serum tests in identifying M proteins

**Figure 3:**
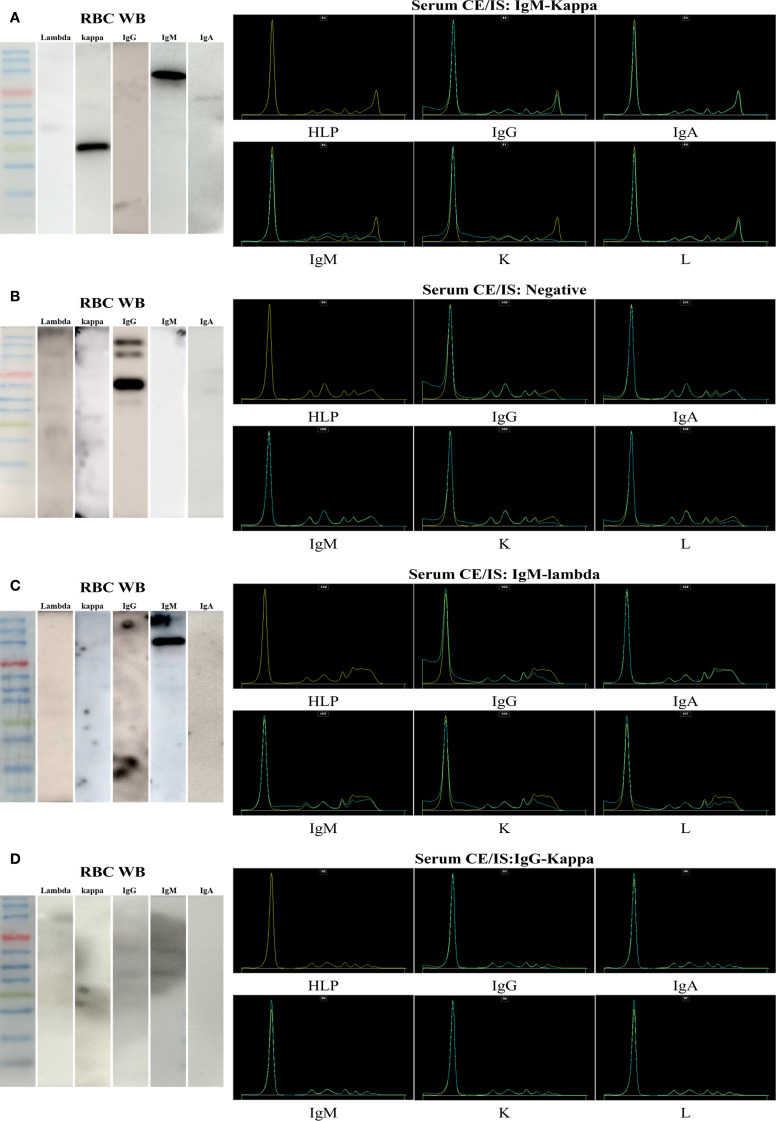
Representative comparisons of erythrocyte electrophoresis and serum CE/IS results. The left side of each image displays the Western blot results of erythrocyte membrane proteins, while the corresponding right side presents the results of serum CE/IS. **(A)** The results from a patient showing concordance between erythrocyte Western blot and serum CE/IS. **(B)** The results from a patient with negative serum CE/IS, yet demonstrating extrinsic monoclonal immunoprotein bands on erythrocytes. **(C)** The results from a patient exhibiting abnormal and inconsistent findings between serum CE/IS and erythrocyte electrophoresis. **(D)** The results from a patient with abnormal serum CE/IS but negative erythrocyte electrophoresis.

Among the 49 patients with monoclonal gammopathy enrolled in this study, 45 (91.8%) had M proteins detected on RBCs, 38 (77.5%) had abnormal CE/IS results, 36 (73.5%) had abnormal sFLC ratios, and 32 (65.3%) had abnormal sTLC ratios. When considering the combination of tests for identifying M proteins, the combination of serum tests, including CE/IS, sTLC, and sFLC ratios, could diagnose 98.0% of cases, while the combination of serum tests with erythrocyte electrophoresis could diagnose 100.0% of cases in this study. Furthermore, due to the relatively clean background, the results of erythrocyte electrophoresis appeared to be more recognizable than those of CE/IS. The findings from erythrocyte electrophoresis and serum CE/IS for all patients included in the study are presented in **Supplementary Figure 2**, with representative examples illustrated in **Figure 3**.

As presented in **Supplementary Table 1**, among the 49 patients, the findings from erythrocyte Western blot, serum CE/IS, sFLC, renal biopsy, and bone marrow flow cytometry were concordant in 26 (53.1%) cases (patients 1–26). Eleven (22.4%) of the 49 patients (patients 27–37) demonstrated negative results in CE/IS testing, while erythrocyte Western blot analysis disclosed the presence of extrinsic monoclonal immunoprotein bands, which were consistent with the results of sFLC, bone marrow flow cytometry, or renal histopathology. Patient 32 merits particular attention. She presented with a normal sFLC ratio and negative CE/IS results. Immunofluorescence analysis of the renal biopsy specimen demonstrated deposits of IgG 3+, kappa 3+, lambda weakly positive (±), C3 2+, and C1q 3+, with IgG3 1+ and IgG1/2/4 negative. Light microscopic examination revealed histological features consistent with membranoproliferative and nodular glomerular alterations. Electron microscopic analysis uncovered fine granular electron-dense deposits on the endothelial aspect of the glomerular basement membrane. The final diagnosis considered MGRS (light/heavy chain deposition disease), with the possibility of PGNMID not being definitively excluded. Interestingly, erythrocyte Western blot analysis revealed the presence of extrinsic IgG bands in the absence of kappa or lambda chains, suggesting the deposition of IgG heavy chains on erythrocytes. Eight (16.3%) of the 49 patients (patients 38–45) presented abnormal yet discordant results in serum CE/IS and erythrocyte electrophoresis. Patient 39 merits particular attention. She had normal sFLC ratios; the serum CE/IS result indicated the presence of IgM-lambda, yet erythrocyte electrophoresis analysis revealed distinct and intense bands for IgM in the absence of either kappa or lambda chains. Immunofluorescence analysis of the renal biopsy specimen demonstrated deposits of IgG 1+, IgM 1+-2+, kappa 1+, and lambda 1+. On light microscopic examination, the histological features were indicative of FSGS. Unfortunately, no glomeruli were available for electron microscopy examination, precluding a definitive diagnosis of heavy chain deposition disease. Four (8.2%) of the 49 patients had no or polyclonal immunoproteins on RBCs.

## 4 Discussion

In the present study, we first directly demonstrated the existence of monoclonal proteins binding RBCs in peripheral blood. Our findings showed that not only intact monoclonal immunoglobulins but also monoclonal light or heavy chains were present on the surface of RBCs. Since normal serum polyclonal immunoglobulins rarely bind to RBCs, using RBC proteins for electrophoresis appears to be more sensitive and recognizable than using plasma for electrophoresis to identify M protein and its isotype.

In the closing decades of the twentieth century, several studies discussed the presence of red cell-bound immunoproteins. In healthy individuals, RBC subpopulations fractionated based on density differences revealed a correlation between cellular senescence and the increased binding of trace amounts of IgG, IgM, and IgA bindings. The increasing immune-protein binding with age may be related to the loss of sialic acid and other components from the cell membrane with the appearance of neoantigens. This process was hypothesized to be part of the homeostatic mechanism for removing senescent RBCs (21, 22). In patients with hemoglobin and RBC membrane abnormalities, increased amounts of IgG were present on the older RBCs, as in the normal individual (23). In patients with autoimmune hemolytic anemia (AIHA) and rheumatic diseases, RBC immunoprotein coating might take the form of immune complexes, since RBCs are known to express receptors for C3b (CR1) and have a key role in the transport and elimination of immune complexes containing IgG and C3b (24, 25). Elevated IgG binding to RBCs has also been detected in a few cases of myeloma patients, which was considered to result from nonspecific adsorption (16). In this study, we utilized Western blot analysis to assess the levels of immunoglobulins on the surface of erythrocytes. Our findings revealed that some of the healthy subjects indeed exhibited minimal levels of immunoglobulins on RBCs. Patients with lupus nephritis demonstrated the presence of polyclonal immunoglobulins, including IgG, IgM, and IgA, on the erythrocytes, although all these individuals have tested negative in direct antiglobulin (Coomb’s) assays. No immunoproteins were detected on RBCs in patients with MN who were positive for serum anti-PLA2R antibodies, indicating that not all abnormal immunoglobulins necessarily bound to RBCs.

In patients with monoclonal gammopathy enrolled in this study, distinct M protein bands, not only intact immunoglobulins but also solely immunoglobulin fractions, were detected on RBCs, which indicated that the binding of M proteins to RBCs might not be dependent on antigen-antibody reactions or Fcγ receptors. It has been reported that RBCs can carry amyloid substances, such as amyloid β in Alzheimer’s disease and amylin in type 2 diabetes (13–15). However, we did not detect ApoE, which was often co-deposited in AL amyloidosis, on the surface of RBCs in any patients with AL amyloidosis, suggesting that light chains did not bind to RBCs in the form of amyloid substances. In our study, some patients had M protein on RBCs despite having low levels of circulating free light chains or associated immunoglobulins. The binding of M proteins to RBCs seemed not to be correlated with the serum concentration of M proteins. The binding might be related to aberrant structural properties of the M proteins, which needs to be determined in future investigations.

In our study, erythrocyte electrophoresis seemed to be more sensitive than either sFLC ratio, sTLC ratio, or CE/IS to identify M protein. Furthermore, 53.1% of patients with monoclonal gammopathy had concordant results between erythrocyte Western blot and serum CE/IS. 22.4% of patients had negative serum CE/IS results but extrinsic monoclonal immunoprotein bands on RBCs, which were consistent with the findings from sFLC, bone marrow flow cytometry, or renal histopathology. That is, at least in 75.5% of the patients, the M proteins identified by erythrocyte electrophoresis were accurate. 16.3% of patients had abnormal but inconsistent results between serum CE/IS and erythrocyte electrophoresis. Owing to the subjectivity involved in the interpretation of CE/IS and the complexities associated with the pathological diagnosis of MGRS, it remained inconclusive as to which method, CE/IS or erythrocyte electrophoresis, yielded more accurate results. Although several studies have reported that the sensitivity and specificity of CE/IS and immunofixation electrophoresis were comparable (26–28), our study was limited to serum CE/IS and did not include serum and urine immunofixation results. The comparison of the sensitivity, specificity, and accuracy between RBC electrophoresis and current serological assays for the diagnosis of M proteins also requires further validation through studies with larger cohorts.

There are some limitations of our study. Firstly, the sample size of this study is relatively small, and a larger sample is needed to confirm the role of RBC electrophoresis in the diagnosis of M protein. Secondly, the electrophoretic analysis of erythrocyte membrane proteins is excessively laborious, rendering it impractical for widespread clinical implementation. We have attempted to utilize flow cytometry on six patients with RBC-bound M protein confirmed by Western blot. Regrettably, we were only able to identify a monoclonal kappa light chain on RBCs by flow cytometry in a single instance within a case of kappa-type LCDD (data not shown). The etiology of this discrepancy warrants additional investigation to ascertain whether it is attributable to issues with antigenic epitopes or to the loss of protein during the experimental process. Third, this is a preliminary study; the mechanisms underlying the interaction between M protein and RBCs, as well as the consequences of this binding, warrant further investigation.

## 5 Conclusions

Monoclonal gammopathy disorders are inherently complex and pose significant diagnostic challenges. This study has uncovered the presence of monoclonal immunoglobulins on erythrocytes, which may serve as a supplementary diagnostic indicator for the detection of M protein. The exploration of efficient methodologies for the detection of monoclonal immunoglobulins bound to RBCs, such as flow cytometry or MALDI mass spectrometry, might represent a significant avenue for future research.

## Data Availability

The original contributions presented in the study are included in the article/**Supplementary Material**. Further inquiries can be directed to the corresponding authors.
