# Supplementary material for: Monoclonal immunoglobulins on red blood cells: a potential supplementary diagnostic indicator for monoclonal gammopathies
Source: Front Immunol. 2025 Sep 12;16:1636162. doi: 10.3389/fimmu.2025.1636162 (PMC12463922; doi:10.3389/fimmu.2025.1636162)
Supplement: Supplementary file 1 [file DataSheet1.pdf]

Supplementary Material

1 Supplementary Figures and Tables

1.1 Supplementary Figures

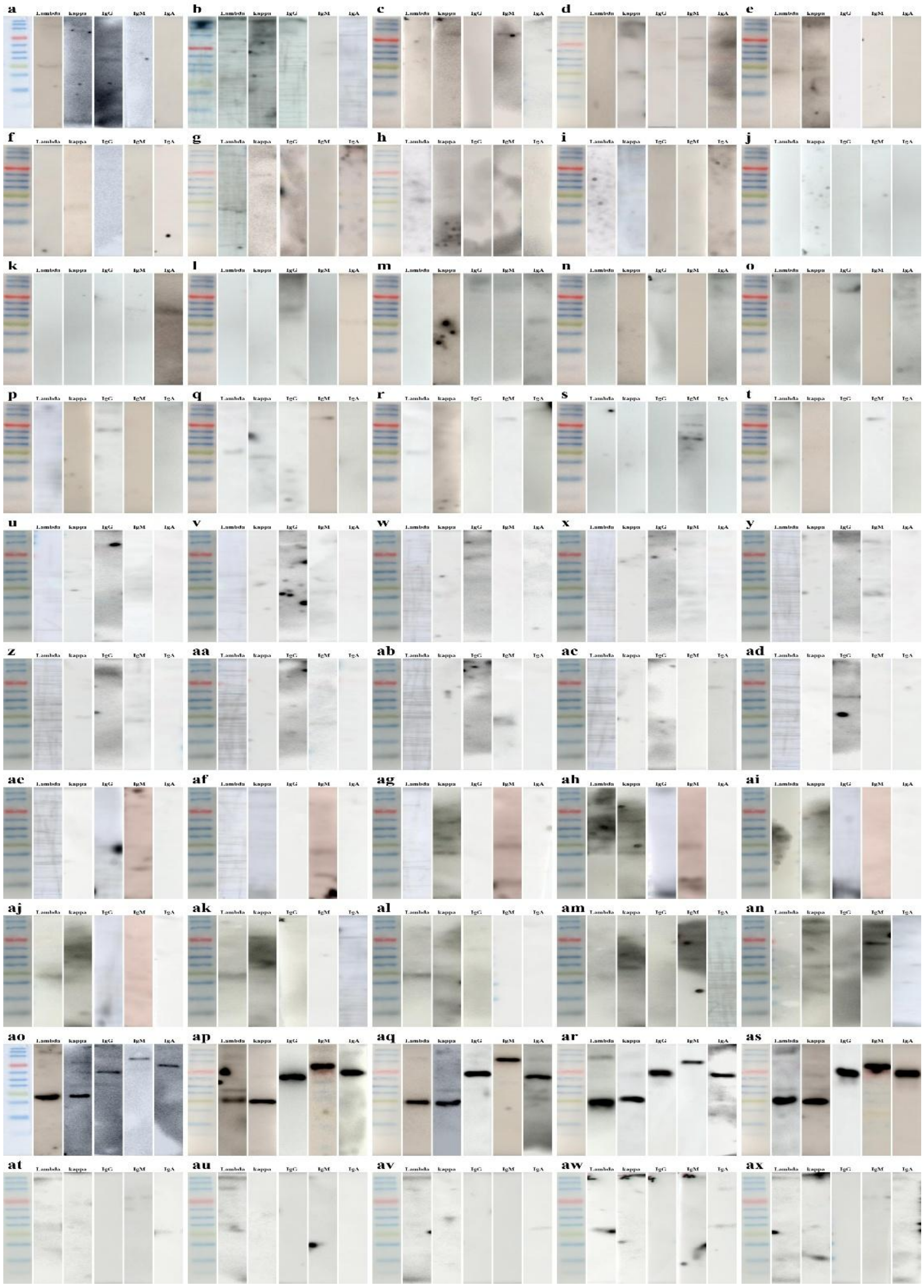

Supplementary Figure 1. The results of erythrocyte electrophoresis of 40 healthy controls, 5 patients with LN, and 5 patients with MN who were positive for anti-PLA2R antibodies. (a-an) Western blot results from healthy controls. (ao-as) Western blot result from 5 patients with LN, showing distinct bands for IgG, IgM, IgA, kappa, and lambda. (at-ax) Western blot result from 5 patients with MN who tested positive for anti-PLA2R antibodies, in which no immunoglobulin was detected on erythrocytes.

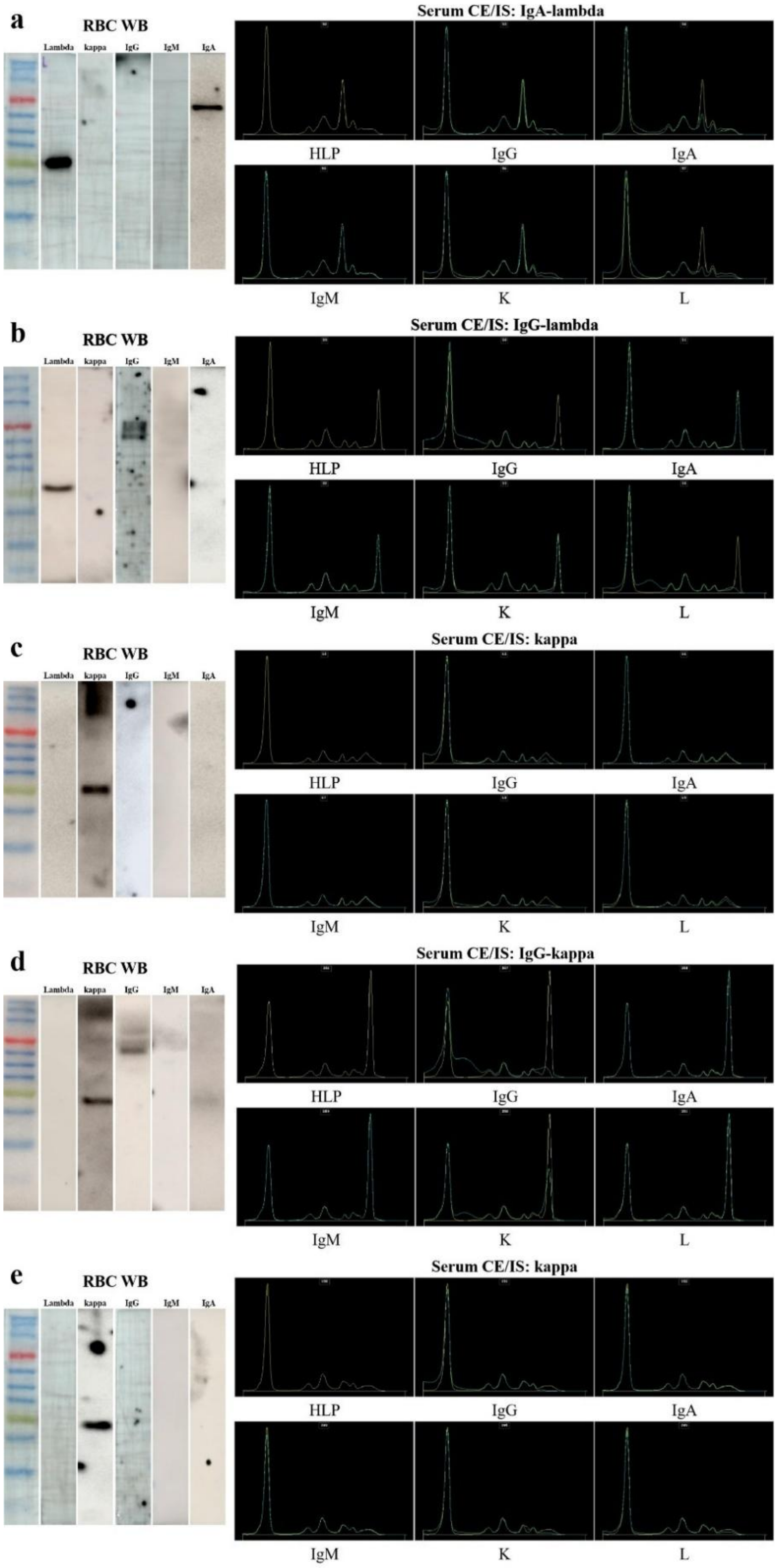

Supplementary Figure 2. The comparisons of erythrocyte electrophoresis and serum CE/IS results of the 49 patients with monoclonal gammopathy. The left side of each image displays the Western blot results of erythrocyte membrane proteins, while the corresponding right side presents the results of serum CE/IS.

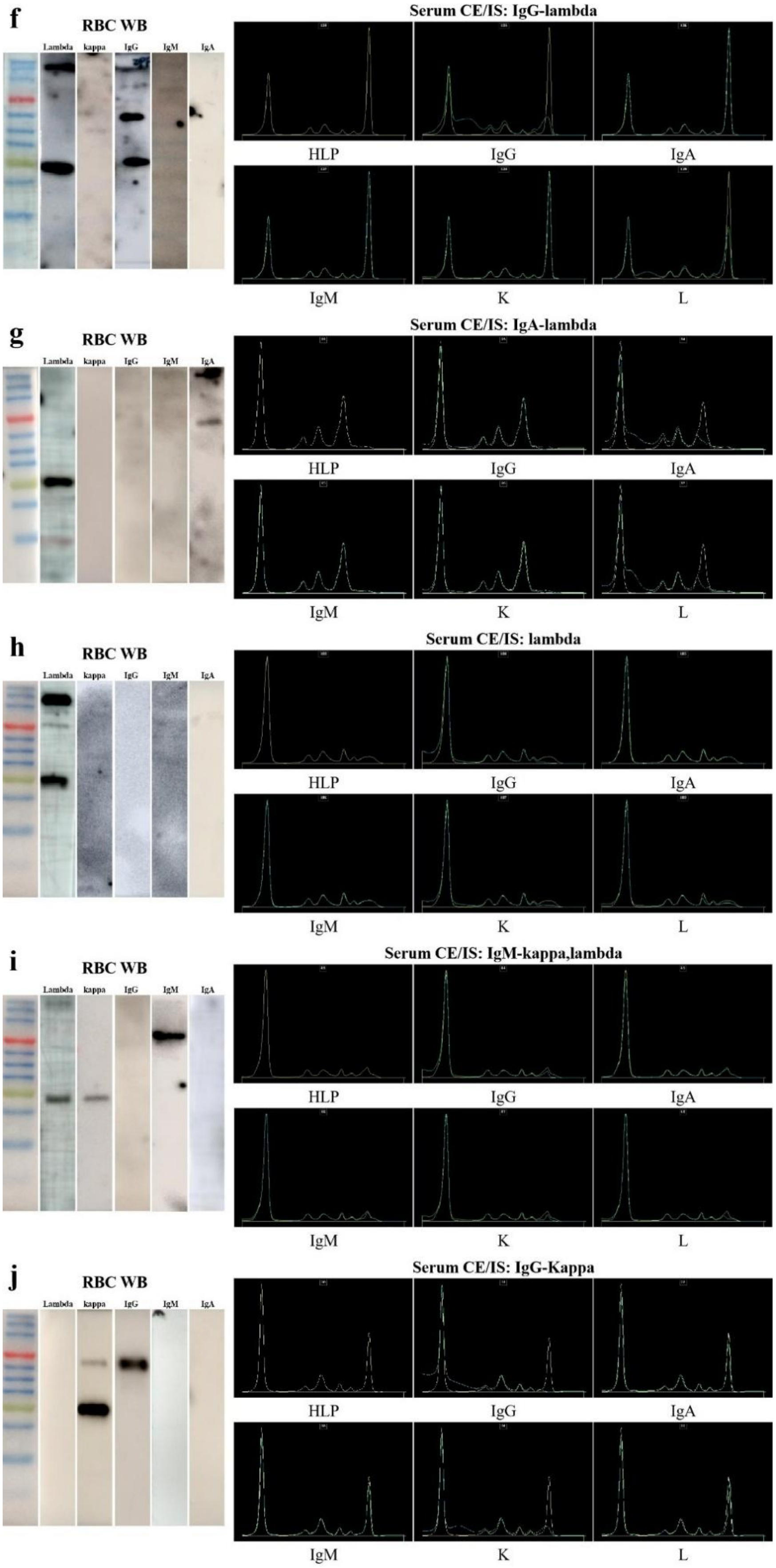

Supplementary Figure 2. The comparisons of erythrocyte electrophoresis and serum CE/IS results of the 49 patients with monoclonal gammopathy. (continued)

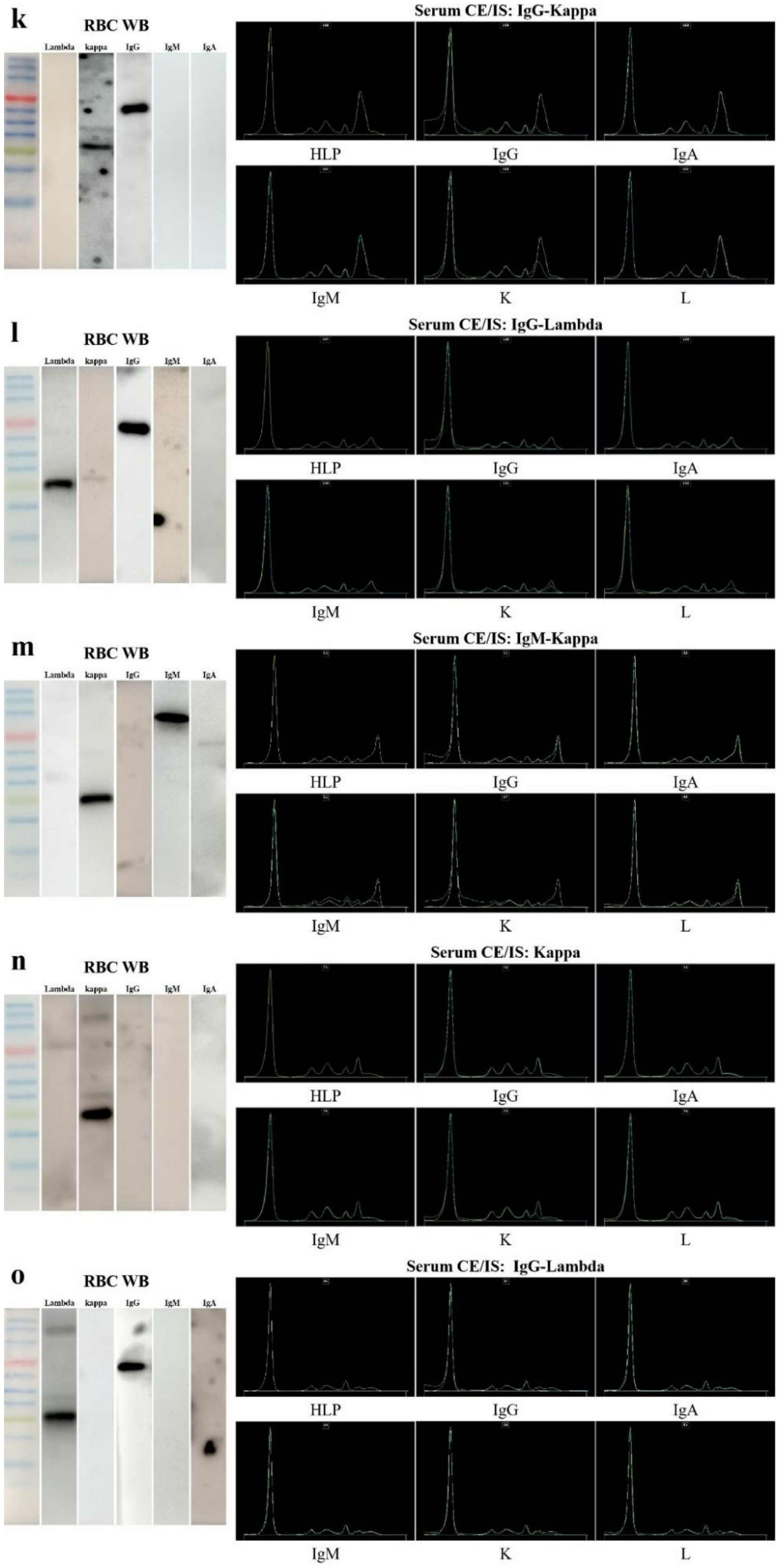

Supplementary Figure 2. The comparisons of erythrocyte electrophoresis and serum CE/IS results of the 49 patients with monoclonal gammopathy. (continued)

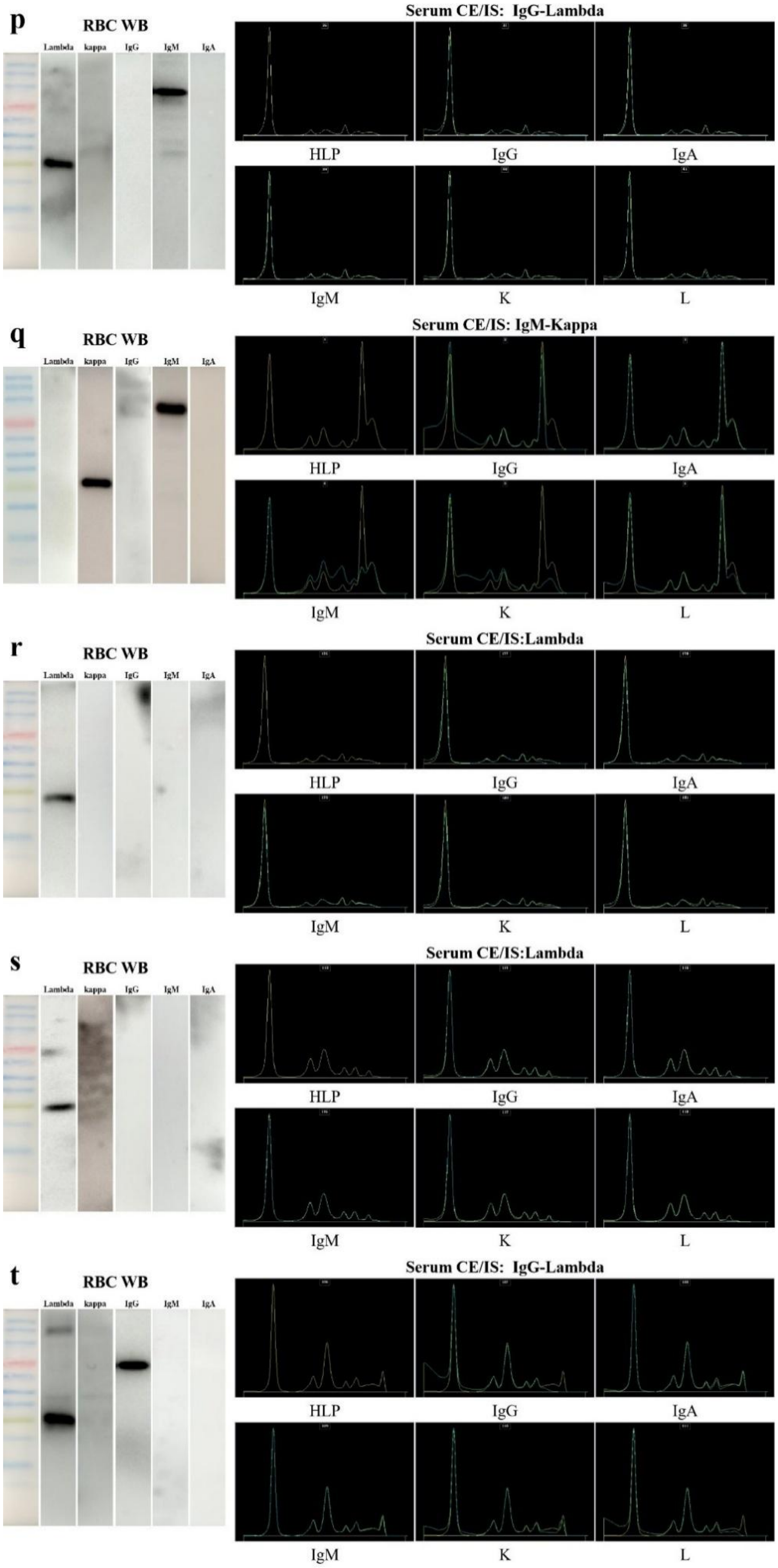

Supplementary Figure 2. The comparisons of erythrocyte electrophoresis and serum CE/IS results of the 49 patients with monoclonal gammopathy. (continued)

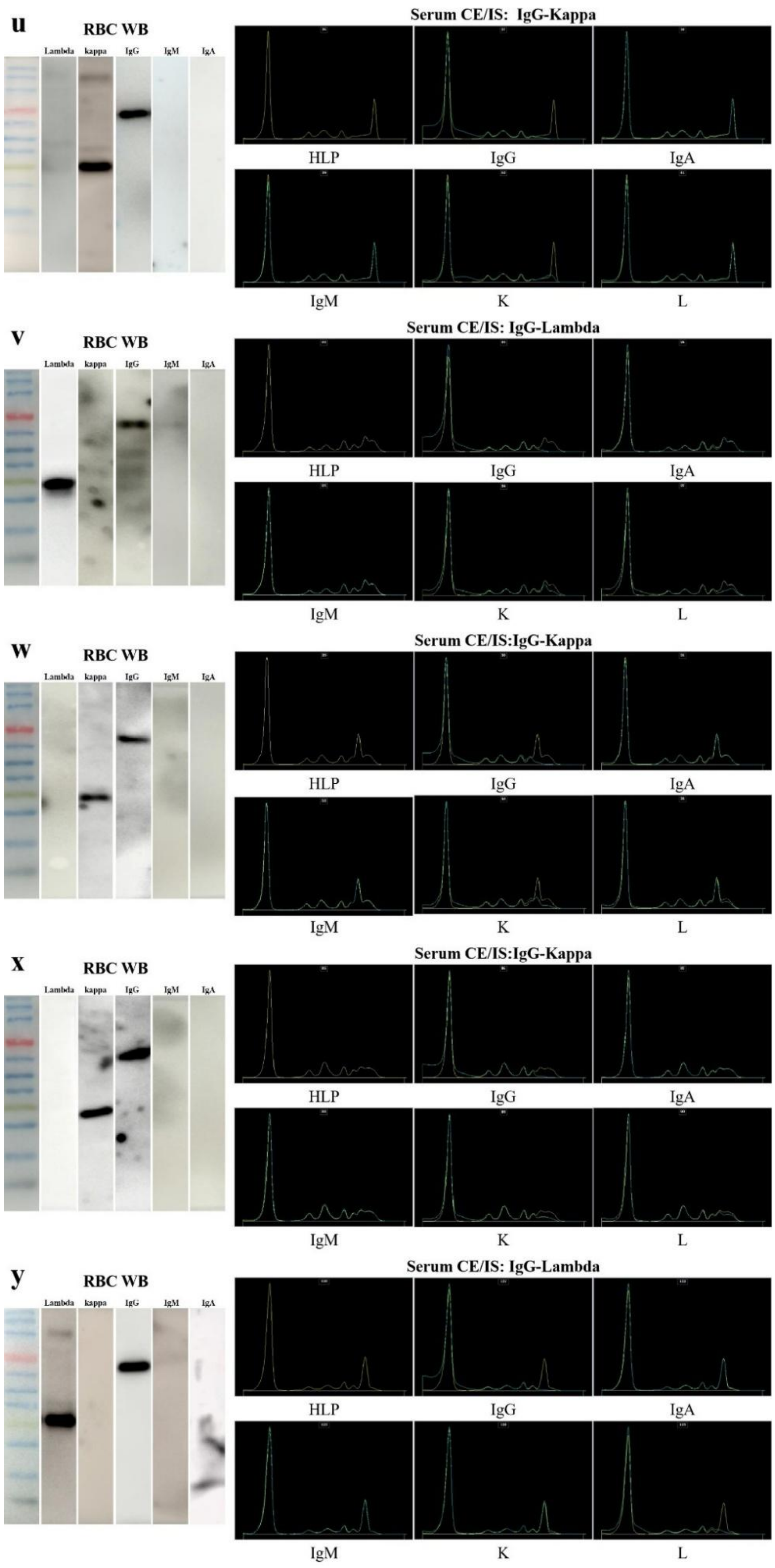

Supplementary Figure 2. The comparisons of erythrocyte electrophoresis and serum CE/IS results of the 49 patients with monoclonal gammopathy. (continued)

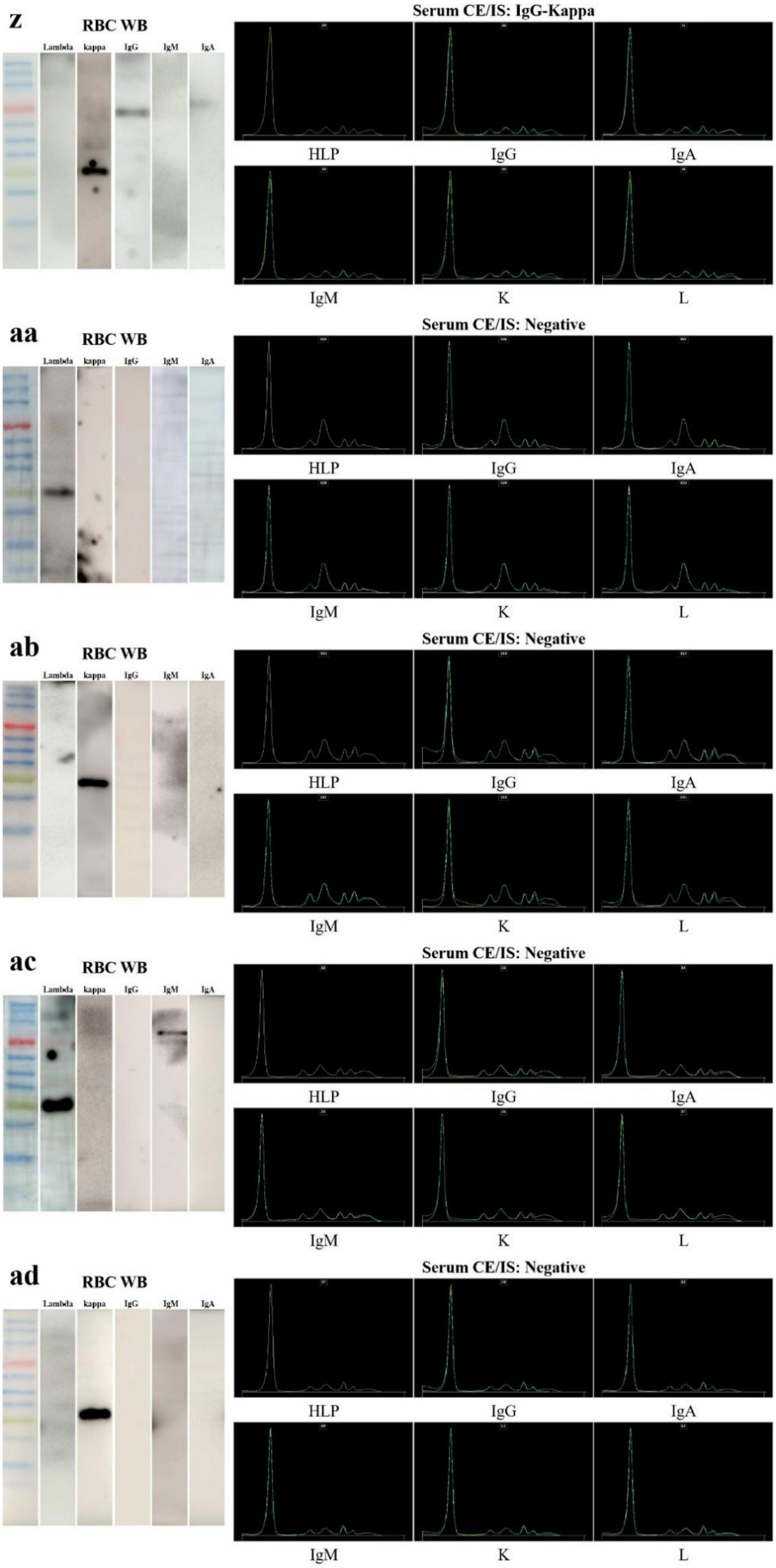

Supplementary Figure 2. The comparisons of erythrocyte electrophoresis and serum CE/IS results of the 49 patients with monoclonal gammopathy. (continued)

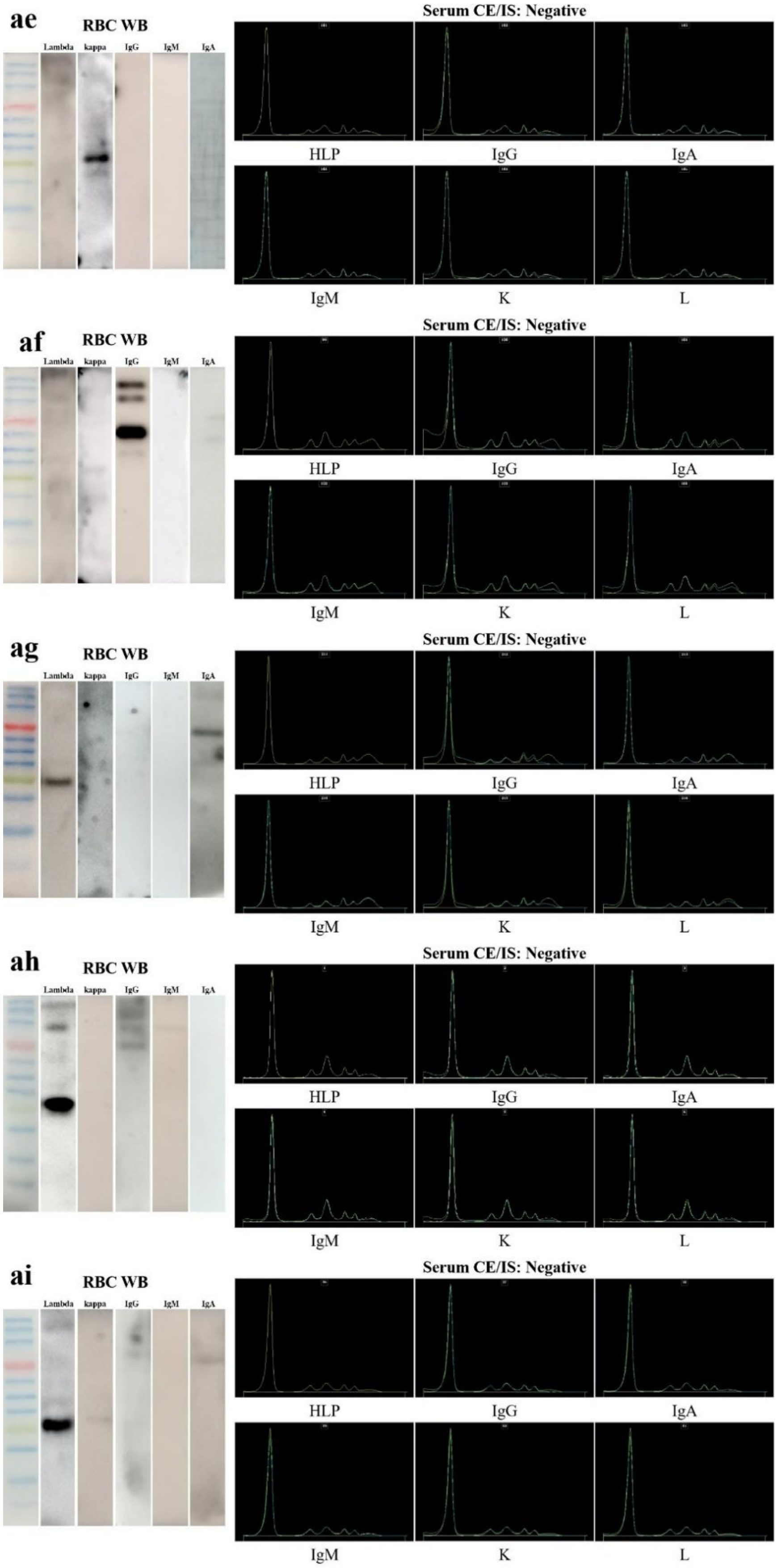

Supplementary Figure 2. The comparisons of erythrocyte electrophoresis and serum CE/IS results of the 49 patients with monoclonal gammopathy. (continued)

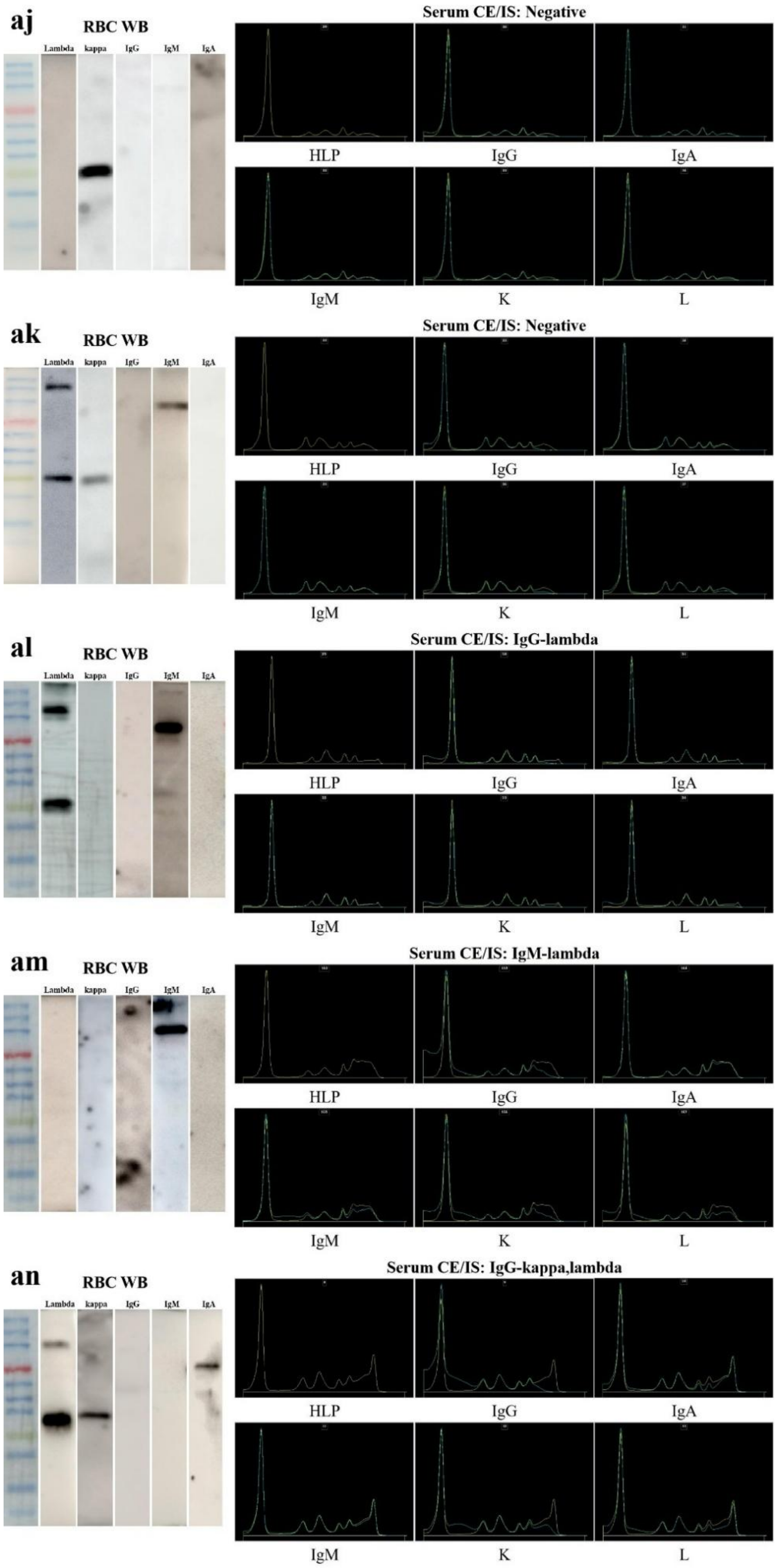

Supplementary Figure 2. The comparisons of erythrocyte electrophoresis and serum CE/IS results of the 49 patients with monoclonal gammopathy. (continued)

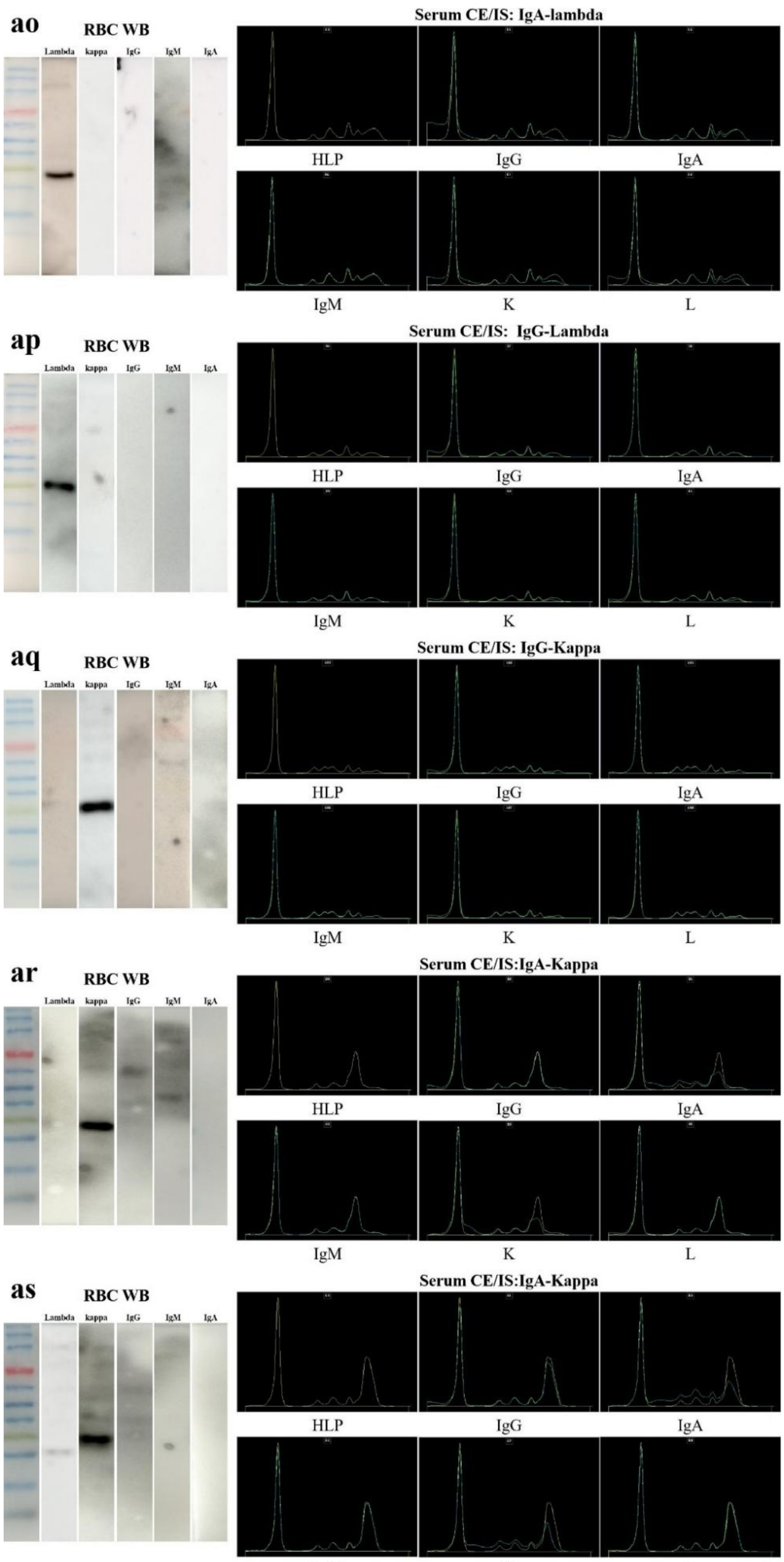

Supplementary Figure 2. The comparisons of erythrocyte electrophoresis and serum CE/IS results of the 49 patients with monoclonal gammopathy. (continued)

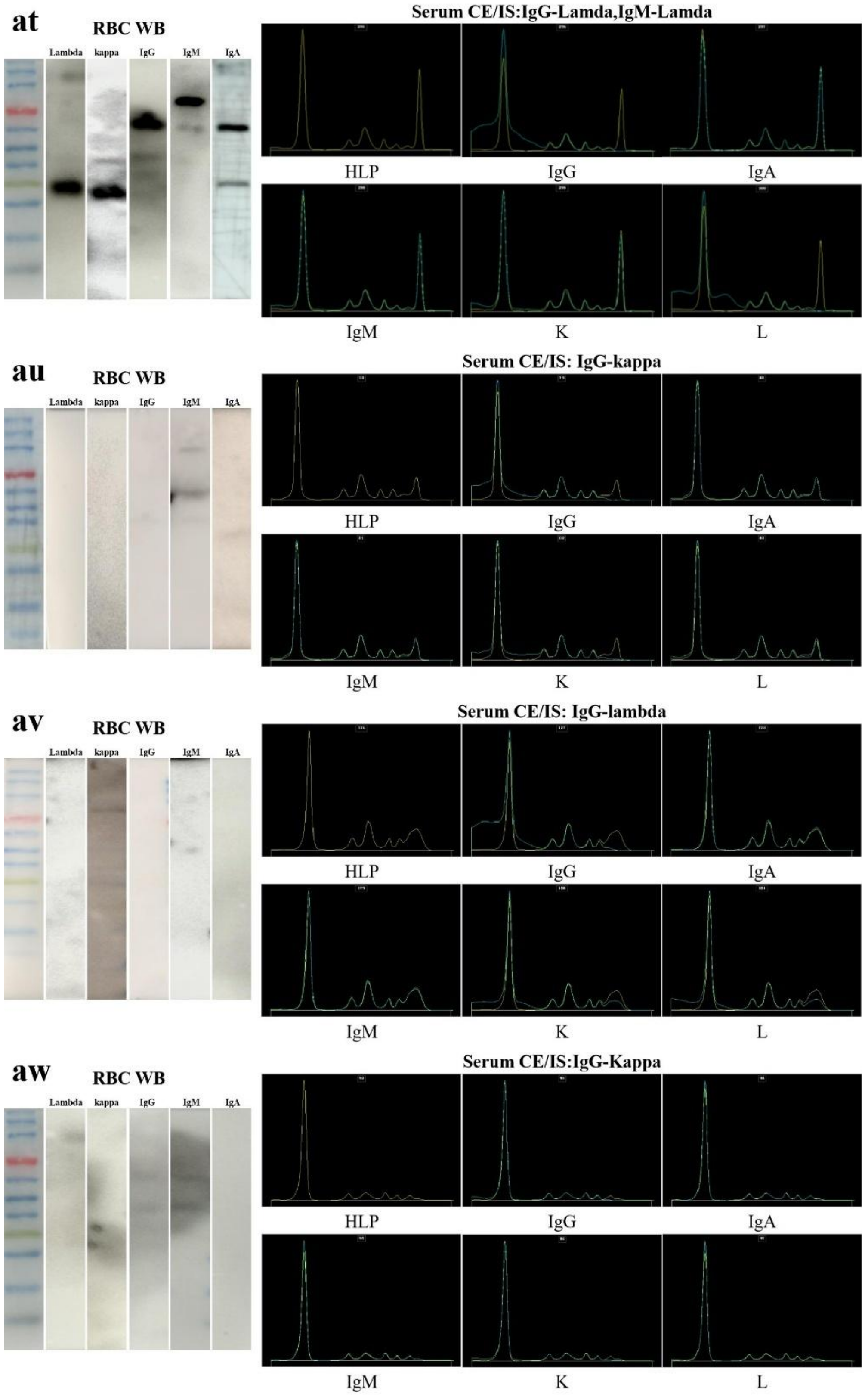

Supplementary Figure 2. The comparisons of erythrocyte electrophoresis and serum CE/IS results of the 49 patients with monoclonal gammopathy. (continued)

1.2 Supplementary Tables

Supplementary Table 1. The findings from erythrocyte Western blot, serum CE/IS, sFLC, renal biopsy, and bone marrow flow cytometry in the 49 patients with monoclonal gammopathy

Patient	RBC WB	CE/IS	κFLC (mg/l)	λFLC (mg/l)	κFLC/ λFLC	Disease	Renal pathology	Abnormal cell clone in bone marrow by flow cytometry
1	IgA (λ)	IgA (λ)	29.1	34.3	0.8	MGRS	AL amyloidosis (λ)	CD38+, CD138+, clambda+ (0.3%)
2	IgG (λ)	IgG (λ)	26.4	822	0.03	MM	LCDD	CD38+, CD138+, clambda+ (5.8%)
3	κ	κ	8460	25.1	337.1	MM	Cast nephropathy	CD38+, CD138+, ckappa+ (17.7%)
4	IgG (κ)	IgG (κ)	10500	76.3	137.6	MM	Not done	CD38dim+, CD138+, ckappa+ (33.8%)
5	κ	κ	8210	49	167.6	MM	Cast nephropathy	CD38+, CD138+, ckappa+ (11.8%)
6	IgG (λ)	IgG (λ)	67.1	1140	0.1	MM	Not done	CD138+, CD 38+, clambda+ (40.1%)
7	IgA (λ)	IgA (λ)	19.1	579	0.03	MM	AL amyloidosis (λ)	CD38+, CD138+, clambda+ (2.1%)
8	λ	λ	11.1	236	0.05	MM	Not done	CD38+, CD138+, clambda+ (8.1%)
9	IgM, κ, λ	IgM (κ), λ	44.9	653	0.1	MM	Not done	CD138+, CD 38+, clambda+ (3.3%)
10	IgG (κ)	IgG (κ)	103	21.4	4.8	MGRS	AL amyloidosis (κ)	CD138+, CD38+, ckappa+ (1.4%)
11	IgG (κ)	IgG (κ)	16.5	13.1	1.3	MGUS	Not done	CD38+, ckappa+ (1.14%)
12	IgG (λ)	IgG (λ)	10.2	20.2	0.5	MM	Not done	CD138+, CD38+, clambda+ (8.7%)
13	IgM (κ)	IgM (κ)	8.7	6.47	1.3	WM	Not done	CD138+, CD38+, ckappa+ (23%)
14	κ	κ	1700	11.2	1517.9	MM	Not done	CD138+, CD38+, ckappa+ (11.2%)
15	IgG (λ)	IgG (λ)	7.8	670	0.01	MM	Not done	CD138+, CD38+, clambda+ (30.9%)
16	IgM (λ)	IgM (λ)	34.1	90.6	0.4	MGRS	PGNMID	Negative
17	IgM (κ)	IgM (κ)	83.8	44	1.9	WM	Not done	CD138+, CD38+, ckappa+(1.3%)
18	λ	λ	13.3	2270	0.01	MM	Not done	CD138dim+, CD38dim+, ckappa+ (12.5%)
19	λ	λ	9.9	617	0.02	MM	Not done	CD138+, CD38+, clambda+ (21.6%)
20	IgG (λ)	IgG (λ)	45.7	173	0.3	MGUS	MN	CD138+, CD38+, clambda+ (0.7%)
21	IgG (κ)	IgG (κ)	71.4	16.5	4.3	MM	Cryoglobulinemia	CD138+, CD38+, ckappa+ (20%)
22	IgG (λ)	IgG (λ)	43.4	43	1.0	MM	Not done	CD138+, CD38dim+, clambda+ (14.9%)
23	IgG (κ)	IgG (κ)	148	64.2	2.3	MGRS	PGNMID	CD138+, CD38+, ckappa+ (0.3%)
24	IgG (κ)	IgG (κ)	13.8	12.7	1.1	MM	Not done	CD138+, CD38+, ckappa+ (10.1%)
25	IgG (λ)	IgG (λ)	5.7	24.9	0.2	MM	Not done	CD138+, clambda+ (17.2%)
26	IgG (κ)	IgG (κ)	5.2	9.7	0.5	MM	Not done	CD138+, ckappa+ (9.7%)

27	λ	Negative	26.1	142	0.2	MM	AL amyloidosis (λ)	CD38+, CD138+, clambda+ (0.4%)
28	κ	Negative	231	34	6.8	MGRS	AL amyloidosis (κ)	CD38+, CD138+, ckappa+ (0.1%)
29	IgM (λ)	Negative	12.8	275	0.05	MM	Not done	CD138+, CD 38+, clambda+ (0.9%)
30	κ	Negative	144	7.2	20.0	MM	LCDD	CD138+, CD38+, ckappa+ (4.8%)
31	κ	Negative	386	15.2	25.4	MM	AL amyloidosis (κ)	CD138+, CD38+, ckappa+ (2.3%)
32	IgG	Negative	51.2	77.4	0.7	MGRS	Light/heavy chain deposition disease, can’t exclude PGNMID	Not done
33	λ, IgA	Negative	24	91.1	0.3	MM	Not done	Negative
34	λ	Negative	21	319	0.1	MGRS	AL amyloidosis (λ)	CD138+, CD38+, clambda+ (0.3%)
35	λ	Negative	3.5	248	0.01	MM	Not done	CD138+, CD38+, clambda+ (11.5%)
36	κ	Negative	43.1	7.8	5.5	MM	Not done	CD138+, CD38+, ckappa+ (7.8%)
37	IgM, κ, λ	Negative	27	602	0.05	MGRS	LCDD	Negative
38	IgM (λ)	IgG (λ)	5.9	606	0.01	MGRS	AL amyloidosis (λ)	CD38+, clambda+ (0.7%)
39	IgM	IgM (λ)	48.7	83.5	0.6	MGUS	FSGS	Negative
40	IgA, κ, λ	IgG (κ), λ	175	163	1.1	MM	Not done	CD138+, CD38+, ckappa+ (20.5%)
41	λ	IgA (λ)	22.9	79.9	0.3	MGRS	AL amyloidosis (λ)	CD138+, CD38dim+, clambda+ (0.2%)
42	λ	IgG (λ)	9.9	266	0.04	MM	Not done	CD138+, CD38+, clambda+ (10.5%)
43	κ	IgG (κ)	27.7	33.4	0.8	MM	LCDD	CD138+, ckappa+ (10.2%)
44	κ	IgA(κ)	390	15.8	24.7	MM	Not done	CD138+, CD38+, ckappa+ (5.7%)
45	κ	IgA(κ)	835	10.9	76.6	MM	Not done	CD138+, CD38+, ckappa+ (11.9%)
46	κ, λ, IgG, IgM, IgA	IgG (λ), IgM (λ)	7.6	213	0.04	MGRS	AL amyloidosis (λ)	Not done
47	Negative	IgG (κ)	47.4	29.1	1.6	MGRS	LCDD	CD138+, CD38+, ckappa+ (0.3%)
48	Negative	IgG (λ)	55.1	112	0.5	MGUS	MCD	Negative
49	Negative	IgG (κ)	27	4.4	6.1	MM	Not done	CD138+, CD38+, ckappa+ (7.7%)

RBC, red blood cells; WB, Western blot; CE/IS, capillary electrophoresis and immunosubtraction; κFLC, serum free kappa chain; λFLC, serum free lambda chain; Ig, immunoglobulin; MGRS, monoclonal gammopathy of renal significance; AL amyloidosis, light-chain amyloidosis; MM, multiple myeloma; LCDD, light chain deposition disease; MGUS, monoclonal gammopathy of uncertain significance; WM, Waldenström macroglobulinemia; PGNMID, proliferative glomerulonephritis with monoclonal IgG deposits; MN, membranous nephropathy; FSGS, focal segmental glomerular sclerosis; MCD, minimal change disease. Reference values: serum κFLC (6.7−22.4 mg/l); serum λFLC (8.3−27 mg/l); serum κFLC/ λFLC (0.31−1.56).
